# Creating a new “reality” for medical education: the Nexus Reality Lab for virtual reality

**DOI:** 10.5195/jmla.2019.784

**Published:** 2019-10-01

**Authors:** Jason Lilly, Kellie N. Kaneshiro, Chelsea Misquith, Brandon Dennett

**Affiliations:** Academic Specialist & Library Systems Manager, Ruth Lilly Medical Library, Indiana University School of Medicine, Indianapolis, IN, jlilly1@iuhealth.org; Assistant Director for Library Technology, Ruth Lilly Medical Library, Indiana University School of Medicine, Indianapolis, IN, kkaneshi@iu.edu; Emerging Technologies Librarian, Ruth Lilly Medical Library, Indiana University School of Medicine, Indianapolis, IN, cmisquit@iu.edu; Nexus Manager, Ruth Lilly Medical Library, Indiana University School of Medicine, Indianapolis, IN, bdennett@iu.edu

## Abstract

**Background:**

The Technology Team at the Ruth Lilly Medical Library, Indiana University (IU), first started exploring virtual reality (VR) in 2016. In 2017, we began offering weekly sessions dubbed VRidays (“VR Fridays”) to give students an opportunity to experience the technology. We also purchased a portable VR setup that allowed us to demonstrate VR at our regional campuses.

**Description:**

To lower the entry barrier to VR, the Technology Team collaborated with the IU Advanced Visualization Lab to establish a reality lab in our collaborative learning space. The lab opened in the fall of 2018 and consists of four high-end VR stations that are accessible to students at any time, but they can also make an appointment for a more guided experience. Information and instructions are available on a LibGuide.

**Conclusion:**

We are currently collecting data on the number of unique users and evaluating application usage. We are working on a feedback mechanism and looking to develop collaborative partnerships across the university.

Virtual reality (VR) has been making inroads into health care for several years, from treating post-traumatic stress disorder (PTSD) and distracting patients from pain to helping future physicians train for surgical procedures. In early 2017, the Technology Team at the Ruth Lilly Medical Library at Indiana University (IU) started exploring VR as an emerging technology for the library’s Nexus Collaborative Learning Lab (hereafter referred to as “Nexus”), a space where students, faculty, and staff can collaborate on projects and share ideas. We later purchased an HTC Vive VR headset and a high-end MSi laptop, nicknamed “the Beast,” to showcase the technology at other campuses. In November 2017, our library systems manager started offering weekly VR sessions dubbed VRidays (“VR Fridays”) for medical students that ran until the summer of 2018.

In May of 2018, our team collaborated with colleagues at the University Information Technology Services (UITS) Advanced Visualization Lab (AVL) to set up a Reality Lab in the Nexus. The AVL agreed to fund approximately $ 1,750 per station, which is roughly half what they would originally cost. Our Reality Lab consists of 4 stations featuring gaming-level MSi towers with HTC Vive headsets. Each is preloaded with a menu of 24 select applications via Steam, a gaming platform offering a variety of VR and non-VR applications. The selected applications vary from medical education programs like 3D Organon VR Anatomy to programs that we consider stress relief for our students like Tilt Brush by Google and Guided Meditation VR. The Reality Lab had its official launch on August 31, 2018, with an open house that was well attended by medical students, staff, and faculty.

A unique aspect of the Reality Lab is that it is in our twenty-four-hour space and is open for medical students to use at any time. Our Nexus manager has an office in the Nexus to better assist users during business hours, and users can request a guided experience from the team via email, if needed. More information about the service is available on our library website.

3D Organon VR Anatomy has had the highest usage of the preloaded apps, which demonstrates that students are using the technology for educational purposes. Our team is trying to increase usage of the Reality Lab by focusing on programming and conducting targeted outreach with faculty and students. We have demonstrations planned for subscription VR apps, such as OssoVR, a surgical training app, and EmbodiedLabs, which provides doctor-patient simulations that help build physicians’ empathy to enhance patient care. We hope that these strategies will enable us to connect with faculty who are interested in using VR in their teaching or research.

**Jason Lilly**, jlilly1@iuhealth.org, Academic Specialist & Library Systems Manager, Ruth Lilly Medical Library, Indiana University School of Medicine, Indianapolis, IN

**Kellie N. Kaneshiro, AHIP**, kkaneshi@iu.edu, https://orcid.org/0000-0001-6459-5902, Assistant Director for Library Technology, Ruth Lilly Medical Library, Indiana University School of Medicine, Indianapolis, IN

**Chelsea Misquith**, cmisquit@iu.edu, https://orcid.org/0000-0002-4274-0264, Emerging Technologies Librarian, Ruth Lilly Medical Library, Indiana University School of Medicine, Indianapolis, IN

**Brandon Dennett**, bdennett@iu.edu, Nexus Manager, Ruth Lilly Medical Library, Indiana University School of Medicine, Indianapolis, IN

